# In Vitro Fermentation Patterns and Methane Output of Perennial Ryegrass Differing in Water-Soluble Carbohydrate and Nitrogen Concentrations

**DOI:** 10.3390/ani10061076

**Published:** 2020-06-22

**Authors:** M. Jordana Rivero, Juan P. Keim, Oscar A. Balocchi, Michael R.F. Lee

**Affiliations:** 1 Departamento de Ciencias Agropecuarias y Acuícolas, Facultad de Recursos Naturales, Universidad Católica de Temuco, Rudecindo Ortega 02950, Temuco 4780000, Chile; jordana.rivero-viera@rothamsted.ac.uk; 2 Rothamsted Research, North Wyke, Okehampton, Devon EX20 2SB, UK; michael.lee@rothamsted.ac.uk; 3 Animal Production Institute, Faculty of Agricultural Sciences, Universidad Austral de Chile, Independencia 641, Valdivia 5090000, Los Ríos, Chile; obalocch@uach.cl; 4 Bristol Veterinary School, University of Bristol, Langford, Somerset BS40 5DU, UK

**Keywords:** high sugar grass, greenhouse gases, nutrient use efficiency, volatile fatty acids, fertilisation rate, defoliation frequency

## Abstract

**Simple Summary:**

Globally, the livestock sector is responsible for 37% of total anthropogenic methane emissions, most of which are produced from enteric fermentation of ruminants. Livestock is also responsible for 65% anthropogenic nitrous oxide and 64% of anthropogenic ammonia emissions. The literature reports several dietary management options to reduce greenhouse gas emissions from ruminants, and potentially improve productivity. However, strategies that aim to reduce the emissions of one specific greenhouse gas can have side effects (increase) on other pollutant gases. In this study, we evaluated the effect of two types of perennial ryegrass (PRG) pastures differing in their concentration of water-soluble carbohydrates (WSC, high (HS) and low (LS)) on the in vitro nitrogen use efficiency in the rumen and on methane emissions. The greater WSC and lower crude protein (CP) concentrations of high sugar pastures modified in vitro rumen fermentation, tending to increase total volatile fatty acids (VFA) production, reduce acetate:propionate ratio and methane (CH_4_) concentration, and improve nitrogen (N) use efficiency through lower rumen ammonia-N (NH_3_-N) concentrations. In vivo studies with cattle are required to confirm the potential of these measures to increase the sustainability and reduce the environmental impact of grazing livestock production systems.

**Abstract:**

The objective of this study was to determine the effect of perennial ryegrass (PRG) forages differing in their concentration of water-soluble carbohydrates (WSC) and crude protein (CP), and collected in spring and autumn, on in vitro rumen fermentation variables, nitrogen (N) metabolism indicators and methane (CH_4_) output, using a batch culture system. Two contrasting PRG pastures, sampled both in autumn and spring, were used: high (HS) and low (LS) sugar pastures with WSC concentrations of 322 and 343 g/kg for HS (autumn and spring), and 224 and 293 g/kg for LS in autumn and spring, respectively. Duplicates were incubated for 24 h with rumen inocula in three different days (blocks). Headspace gas pressure was measured at 2, 3, 4, 5, 6, 8, 10, 12, 18, and 24 h, and CH_4_ concentration was determined. The supernatants were analysed for individual volatile fatty acids (VFA) concentrations, and NH_3_-N. The solid residue was analysed for total N and neutral detergent insoluble N. Another set of duplicates was incubated for 4 h for VFA and NH_3_-N determination. The HS produced more gas (218 vs. 204 mL/g OM), tended to increase total VFA production (52.0 mM vs. 49.5 mM at 24 h), reduced the acetate:propionate ratio (2.52 vs. 3.20 at 4 h and 2.85 vs. 3.19 at 24 h) and CH_4_ production relative to total gas production (15.6 vs. 16.8 mL/100 mL) and, improved N use efficiency (22.1 vs. 20.9). The contrasting chemical composition modified in vitro rumen fermentation tending to increase total VFA production, reduce the acetate:propionate ratio and CH_4_ concentration, and improve N use efficiency through lower rumen NH_3_-N.

## 1. Introduction

Globally, the livestock sector is responsible for 37% of total anthropogenic methane (CH_4_) emissions, most of which is produced from enteric fermentation by ruminants. Moreover, the sector is responsible for 65% of anthropogenic nitrous oxide (N_2_O) and 64% of anthropogenic ammonia (NH_3_) emissions [1]. Thus, the high level of greenhouse gas (GHG) and atmospheric pollutant emissions gives an opportunity for envisaging mitigation strategies through ruminant sector actions. A reduction in GHG emissions and NH_3_ by cattle would not only reduce their environmental footprint, but also potentially improve productivity, as more feed inputs could potentially be directed to animal production (growth and lactation) [2].

There are many diet management options in the literature reported to reduce CH_4_ emissions from ruminants, such as dietary oils, feeding of nitrates, the urea treatment of dietary straw [3], and supplementation with concentrate diets [4] such as straights or distillers grains. However, such strategies may also increase nitrogen excretion and NH_3_ emissions, leading to an increase in the release of N_2_O [5], therefore offsetting one GHG with another and higher levels of NH_3_.

Particularly in grazing systems, the mitigation of CH_4_ emissions can be achieved by improving the digestibility of forages through grazing management or species selection e.g., the use of high-sugar grasses (HSG) [6]. The hypothesis behind the development of HSG cultivars [7] at the Institute for Grassland and Environmental Research (IGER), UK, was that a high-WSC forage would improve the balance of energy with readily available soluble forage-N for the rumen microbial population, thus improving protein (N)-use efficiency (NUE) in the rumen, via greater conversion of forage-N into microbial-N and lower loss via NH_3_ [8,9]. In addition, the supply of more available energy (WSC) will improve forage digestibility and influence rumen microbial carbohydrate metabolism and, subsequently, volatile fatty acid patterns towards the H-sink propionate, therefore potentially reducing substrate for CH_4_ formation [8].

In pasture-based systems, improving the nutritional quality of the grazed forage is crucial, as supplementation may be more restricted in these systems [10]. However, even though increasing WSC content of pasture has the potential to improve efficiency and reduce GHG emissions, the expression of the high sugar trait has shown inconsistencies when comparing UK and New Zealand trials [11,12]. This could be explained by a genetic by environment (G × E) interaction [12], where temperature plays a fundamental role. Furthermore, pasture management has a significant effect on WSC concentration and WSC to CP ratio. Increasing the defoliation frequency, e.g., going from three leaves per tiller to two leaves per tiller, decreases the WSC concentration, increases the CP concentration, and decreases the WSC to CP ratio [13]. Nitrogen fertilisation rate also affects these components; increases in the amount of N applied per hectare increases CP and decreases WSC concentrations, leading to a decrease in WSC to CP ratio [14]. These findings support the strategy of grazing at the three-leaf stage as a way of improving the WSC to CP ratio and, subsequently, the balance between energy and protein in the rumen.

Recently, Rivero et al. [15] found that the combined management of defoliation frequency and N fertilisation rate has a marked effect on WSC concentration, particularly in early spring, and on CP and WSC to CP ratio in autumn. However, this improvement in pasture nutritional composition has not consistently shown responses in the animal. For instance, Miller et al. [16] reported an increase in true protein in milk and a decrease in urinary N excretion of dairy cows fed HSG, while Tas et al. [17] found no difference in N utilisation nor milk protein content. Whereas reports on the effect of HSG on ruminal CH_4_ production reveal little consistent responses either in vitro or in vivo on CH_4_ emissions [9,18,19,20]. Studies evaluating the effect of HSG on both CH_4_ production and NUE are scarce. Furthermore, as described above, the response of measures such as HSG, nitrogen fertilisation and leaf stage on WSC, CP concentrations and the WSC to CP ratio varies across the two main grazing seasons. Thus, the effect of these measures on CH_4_ output and NUE may be different in autumn compared to spring.

In vitro cumulative gas production techniques were developed to predict the fermentation of ruminant feedstuffs, measuring gas produced as an indirect indicator of fermentation kinetics [21]. This technique can also provide a measure of the proportion of feed that is fermented, as opposed to that which is partitioned to microbial growth [22], and an estimation of the relative production of CH_4_ among different substrates. Previous studies using this technique have analysed the effect of WSC concentration of forage on CH_4_ production, either by manipulating WSC concentration via addition of external carbohydrates [8,23] measuring the CH_4_ at the end of the incubation period [24], which can lead to contradictory results and does not reflect how the in vitro CH_4_ output evolves throughout the fermentation process. Others have focused on CH_4_ production without assessing the effect on other outputs of the fermentation process, such as VFA production or N metabolism products [25,26].

In the present study, contrasting WSC concentrations were obtained by combining genetics and management, i.e., cultivars differing in their potential for WSC concentration in combination with differing defoliation and N fertilisation rates. These strategies can be routinely applied on a commercial farm. The objective of this study was to determine the effect of PRG forages differing in their concentration of WSC and CP, and collected in spring and autumn, on in vitro rumen fermentation variables, N metabolism indicators and CH_4_ output using a batch culture system. We hypothesised that the combination of cultivar selection and agronomic management (i.e., reduction in N fertilisation with a longer regrowth period) will reduce in vitro CH_4_ production and increase NUE, through modifications in the fermentation patterns.

## 2. Materials and Methods

All animal procedures were performed in accordance with the UK Animals (Scientific Procedures) Act and associated guidelines and, approved by the Animal Ethics Committee of the Universidad Austral de Chile (144/2013).

### 2.1. Pasture Management

A 1-ha plot was ploughed and prepared in August 2015 for sowing two PRG (*Lolium perenne* L.) cultivars in the research farm at La Araucania Region (Chile). Prior to sowing, and following soil tests, the experimental site was fertilised with 120 kg/ha P_2_O_5_, 90 kg/ha of K_2_O. Half of the area was sown with a “high sugar” cultivar (HS, Expo, diploid, late flowering), and the remaining half area with a standard ‘low sugar’ cultivar (LS, Extreme, diploid, intermediate flowering), with both cultivars sown at a rate of 28 kg/ha seed. Apart from the cultivar, pasture treatments differed in agronomic management: the HS pasture was defoliated at the stage of three leaves per tiller, and with a N fertilisation rate equivalent to 83.3 kg/ha N per year, whilst the LS pasture was defoliated at the stage of two leaves per tiller, with a N fertilisation rate equivalent to 250 kg/ha N per year. These contrasting agronomic managements were aimed to boost the difference among pastures regarding WSC and CP concentrations.

### 2.2. Pasture Sampling

During autumn (April 2016) and spring (end of September–early October 2016) plots were grazed with dairy cows, grazing in six daily strips. Forage samples were collected the day before (at noon), the cows were allocated to graze a new strip (after morning milking). Each day, fresh grass pooled samples of circa 150 g per strip were collected to a residual height of 5 cm, from at least five random points within the strip, frozen immediately in liquid N_2_, stored at −20 °C until freeze dried, and then ground through a 1 mm sieve.

Ground forage (6 g DM) from each daily strip (six for each sward type) were pooled together in pairs: strips one and two comprised block 1, strips three and four block 2, and strips five and six block 3. This procedure was followed for both set of samples obtained in autumn and spring. Each block of samples (three for each of the HS and LS plots) represented the field replicate, and were analysed for WSC [27], N content was determined by combustion (Leco Model FP-428 Nitrogen Determinator, Leco Corporation, St Joseph, MI, USA), and was used to calculate CP content (N × 6.25), neutral detergent fibre was determined as aNDF [28] using heat-stable amylase, acid detergent fibre (ADF; AOAC [29]; method 973.18), and ash (AOAC [29]; method 942.05). Hemicellulose was calculated as the difference between aNDF and ADF (these analyses were sequential on the same samples).

### 2.3. In Vitro Incubations

The method described by Theodorou et al. [21] was used for the in vitro incubations. The four treatments (the factorial combination of two sward types and two seasons) from each block were incubated separately from the other blocks, thus the first block was incubated during day one; at day two those corresponding to block number two, and at day three samples from block three.

Duplicates of each field replicate (1 g DM) were combined within 160 mL fermentation bottles with 85 mL of Goering-Van Soest medium and 4 mL of reducing agent at 39 °C under a CO_2_ anaerobic environment with two blanks. The bottles were covered with rubber stoppers and aluminium seals. Rumen fluid (10 mL) was injected into the bottles. The inoculum was obtained from two lactating Holstein-Friesian dairy cows, prepared with rumen cannulas, that were offered 70% of grass pasture and 30% commercial concentrate in parlour with a composition of 187 g/kg DM; 184 g CP/kg DM; 378 g NDF/kg DM in the diet. Rumen fluid was collected before the donor cows were fed, and transported immediately to the laboratory in thermos flasks, pooled, flushed with CO_2_ and then filtered through four layers of cheesecloth. Inoculation was performed one hour after rumen fluid collection. Once incubated, the bottles were placed in a water bath at 39 °C.

Headspace gas pressure was measured with a manual pressure transducer (PCE Instruments, Tobarra, Albacete, Spain) at: 2, 3, 4, 5, 6, 8, 10, 12, 18, and 24 h, and the volume of gas produced was measured by extracting it with a syringe from the fermentation bottle, until the visual display of the transducer read zero. Bottles were agitated manually at every gas reading. After extraction, 5 mL of the gas produced at every incubation time was stored in pre-evacuated tubes (Labco Ltd., Lampeter, UK) for further determination of CH_4_ concentrations in the headspace gas. Fermentations were arrested at 24 h by placing the bottles on ice. Another set of duplicates was incubated for 4 h for VFA and NH3-N determination. Thus, 16 bottles were incubated at each incubation run.

### 2.4. Analyses

The analytical replicates of each incubation were pooled in a pre-weighed bottle that was kept on ice and then centrifuged at 15,000× *g*. The supernatant was decanted and analysed for VFA concentrations using a gas chromatograph equipped with a flame ionisation detector, model 6850 autoinjector (Agilent Technologies, Mississauga, Ontario, Canada), and fitted with a DB-FFAP column (30 m × 250 mm × 25 µm, Agilent Technologies, Mississauga, Ontario, Canada). The NH_3_-N concentration in the supernatant was determined by a colorimetric method [30]. Solid residues were washed with distilled water and recentrifuged further at 15,000× *g* for 30 min. The supernatant was decanted, and the solid residue freeze-dried and weighed prior to analysis. Total N and neutral detergent insoluble N (NDIN) were determined by combustion (Leco Model FP-428 Nitrogen Determinator, Leco Corporation, St Joseph, MI, USA). The CH_4_ content of the headspace was measured by gas chromatography (Perkin Elmer Clarus 580 Gas Chromatograph) fitted with an Elite^®^ PLOT Q mega-bore column, and calibrated with an external standard of known composition (2.05, 5.18, 10.18, 50.1, and 101.2 ppm CH_4_; BOC specialty gases, Wembley, UK).

### 2.5. Calculations

Values of in vitro apparent OM digestibility (OMD), in vitro true OM digestibility (tOMD), in vitro NDF digestibility (NDFD), and partitioning factor (PF) were calculated according to Blümmel et al. [31,32]. Microbial nitrogen (MN) was estimated as the difference between total N and NDIN concentration in the solid residue [33]. Both in vitro N digestibility (ND) and NDFD were corrected for microbial mass, thus representing the in vitro true digestibility. The efficiency of microbial protein synthesis (EMPS) was calculated according to Wales et al. [34], and bacterial NUE was estimated according to Bach et al. [35]. After correcting for gas production (GP) of the blanks, GP data were fitted to the generalised Michaelis-Menten model without a lag phase [36] as:

GP = A × (Tn/(Tn + Kn))(1)

where GP is gas production at time T; A is the asymptote gas production (mL); n is the value determining the shape of the curve; and K is the time to produce half of A. The other parameters were calculated according to Groot et al. [37] and France et al. [38]:

Degradation rate at half-life = n/(2 × K)(2)

Maximal degradation rate = (n − 1)((n − 1)/n)/k(3)

Time to ferment 50% of the substrate = K × ((X/(1 − X))(1/n))(4)

where X = 0.50

The formula for the CH_4_ volume produced at time point x was the same as used by Tavendale et al. [39]:

CH_4_ (mL) at x = (CH_4_%(x) − CH_4_%(x − 1)) × HSP/100 + CH_4_%(x) × GV(x)/100 (5)

where time x equals 2, 3, 4, 5, 6, 8, 10, 12, 18, and 24 h; x − 1 refers to the previous incubation time; HSP refers to the volume (60 mL) of the bottle above the fermentation material; and GV is the gas volume produced in mL.

### 2.6. Statistical Analysis

Data regarding forage nutritional composition, gas production kinetics, N metabolism parameters and CH_4_ concentration and kinetics were analysed by two-way analysis of variance (ANOVA) with sward type (HS vs. LS) × season (spring vs. autumn) as the main factors. The experimental unit corresponded to the type of pasture in each field block. Volatile fatty acids concentration and NH_3_-N proportion at 4 h and 24 h of incubation were analysed by repeated measures ANOVA, with sward type (HS vs. LS) × season (spring vs. autumn) as the main factors and time of incubation as the time factor. The Fisher least significant difference (LSD) was used for the statistical separation of means. All analyses were performed using Genstat 18 (©VSN International Ltd., Hemel Hempstead, UK).

## 3. Results

### 3.1. Forage Nutritional Value and In Vitro Digestibility

As expected, sward type differed in WSC and CP concentrations (Table 1). The high sugar pasture averaged 332 g/kg DM across seasons, whilst LS averaged 259 g/kg DM (*p* < 0.001). Pasture in spring had 16.4% more WSC than in autumn (*p* < 0.01). The opposite trend was observed in CP; LS had 30.4% more CP than HS, and 29.1% more CP in autumn than in spring (*p* < 0.001). These variations of WSC and CP concentration between sward types and seasons resulted in variation in the ratio among these two nutrients: WSC to CP ratio averaged 2.48 for HS and 1.50 for LS, whilst in spring, the ratio was 0.77 units higher than in autumn (*p* < 0.001). For fibre fractions, aNDF and hemicellulose concentrations did not vary between sward types in spring (*p* > 0.05). However, in autumn, LS had 20.4% more NDF and 33.0% more hemicellulose than HS (*p* < 0.001). Acid detergent fibre was slightly higher (10%) in LS than HS (*p* < 0.05), and in spring than in autumn (3.6%; *p* < 0.05). Organic matter digestibility, either apparent (OMd) or true (tOMd), did not vary between sward types nor seasons, averaging 600 g/kg and 930 g/kg for OMd and tOMd, respectively. NDF digestibility did not vary between sward types nor seasons (*p* > 0.05), averaging 777 g/kg. Ash showed the opposite response between sward types across seasons with higher concentrations of HS in autumn and LS in spring.

### 3.2. In Vitro Gas Production

**Figure 1:**
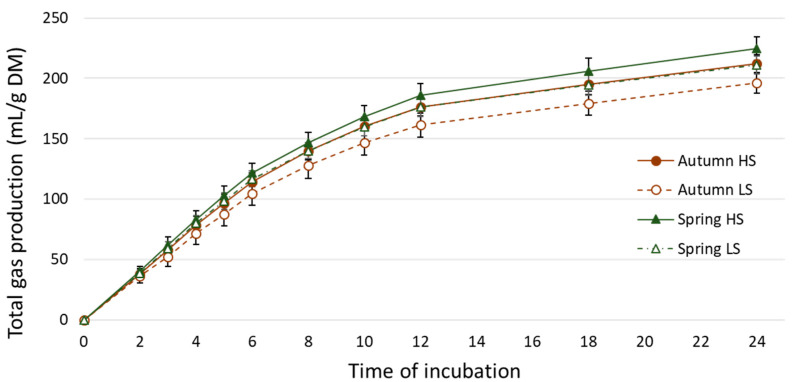
Total gas production throughout the 24 h of in vitro incubation. HS: high sugar pasture; LS: standard cultivar pasture. DM: dry matter.

Total gas production was higher (*p* < 0.05) in HS (218.4 mL/g OM) than in LS (203.6 mL/g OM), and higher in spring (217.9 mL/g OM) than in autumn (204.1 mL/g OM) (Table 2, Figure 1). The partitioning factor did not vary between pasture types (*p* > 0.05) and was higher (*p* < 0.05) in autumn (4.62) than in spring (4.27). The only gas production kinetics parameter that varied was the asymptotic gas production, which was affected by the pasture type (*p* < 0.05), averaging 248.6 mL/g OM for HS and 232.5 mL/g OM for LS (Table 2).

### 3.3. In Vitro Volatile Fatty Acids

There was a significant effect of incubation time on total VFA (tVFA), acetate, propionate, butyrate and branched chain VFA (BCVFA) concentrations, that increased from 4 to 24 h of incubation (*p* < 0.001; Table 3). For the main effects, tVFA concentration tended to be greater (*p* = 0.088) for HS (52.0 mM) than LS (49.5 mM) pasture and tVFA concentrations were greater for spring than autumn pastures with 55.5 and 46.0 mM, respectively. A significant interaction between incubation time and season of the year was observed (*p* = 0.035), and indicated that the difference between autumn and spring pastures in tVFA concentration was increased by 5.9 mM at 4 h and 13 mM at 24 h. Acetate concentration was affected by season (*p* < 0.001), being greater for spring than autumn pastures (36.5 and 30.7 mM, respectively), whereas no significant effects were observed pasture type and all interactions (*p* > 0.05). Concentrations of propionate, butyrate, and BCVFA were affected by pasture type and season of the year (*p* < 0.01), in case of propionate and butyrate, concentrations were always greater for HS and spring compared to LS and autumn pastures, whereas BCVFA concentrations were greater for LS and autumn pastures. In addition, significant ‘incubation time’ × ‘season’ interactions were observed for propionate and butyrate concentrations. Propionate concentration from spring pastures was greater compared to autumn pastures at both 4 h (9.7 and 7.6 mM for spring and autumn, respectively) and 24 h (18.5 and 14.1 mM for spring and autumn, respectively) of incubation; however, the difference across seasons was increased two-fold from 4 to 24 h. In the case of butyrate concentration, the ‘incubation time’ × ‘season’ interaction arose from no differences across seasons at 4 h of incubation, and a greater concentration from fermentation of spring pastures (5.5 mM), compared with autumn (4.5 mM).

The relative proportions of tVFA of acetate and propionate, acetate:propionate (A:P), and (acetate + butyrate):propionate ((A + B)/P) ratios were affected by pasture type (*p* < 0.01). In addition, the relative proportions of propionate and BCVFA (*p* < 0.001), A:P and (A + B)/P ratios (*p* < 0.05) were affected by season. No significant ‘pasture type’ × ‘season’, ‘pasture type’ × ‘incubation time’ and ‘season’ × ‘incubation time’ interactions were observed (*p* > 0.05) for the relative proportion of tVFA of acetate, propionate, butyrate and BCVFA, and A:P and (A + B)/P ratios. Finally, significant ‘pasture type’ × ‘season’ × ‘incubation time’ interactions were observed for propionate relative proportion of tVFA (*p* < 0.01) and (A + B)/P ratio (*p* < 0.05), whereas a trend (*p* = 0.058) was observed for the A:P ratio. Acetate relative proportion of tVFA was greater for LS (67.8 mmol/mol tVFA), compared to HS pastures (65.2 mmol/mol tVFA). The propionate relative proportion of tVFA was greater for HS pastures at 4 h of incubation in both autumn and spring, whereas, at 24 h of incubation, it was greater for HS in autumn, but not different among LS and HS in spring. The butyrate relative proportion of tVFA was only affected by incubation time (*p* < 0.001), being greater at 24 h (7.5 vs. 4.7 mmmol/mol tVFA). The relative proportion of BCVFA tended to be greater (*p* = 0.087) for LS than for HS pastures (2.93 and 2.63 mmol/mol tVFA for LS and HS, respectively), and was significantly greater for autumn (3.3 mmol/mol tVFA) than for spring pastures (2.3 mmol/mol tVFA). Lower A:P and (A + B)/P ratios were observed for HS pastures compared with LS in autumn at 24 h of incubation, whereas there were no differences among pasture type in autumn at 4 h, and autumn and spring at 24 h of incubation.

### 3.4. In Vitro Methane Production and Concentration

**Figure 2:**
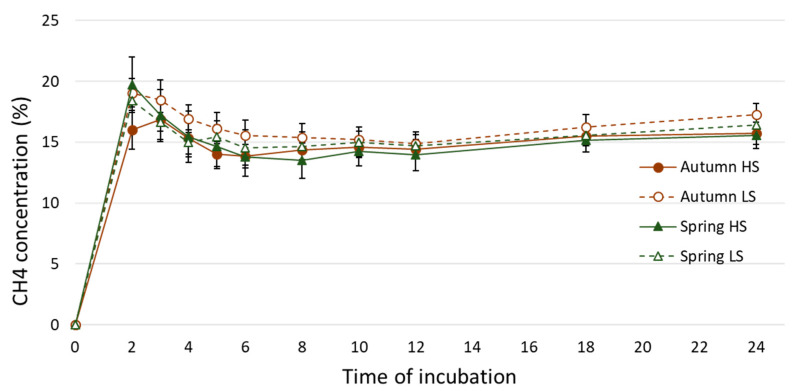
Methane (CH_4_) proportion of total gas production throughout the 24 h of in vitro incubation. HS: high sugar pasture; LS: standard cultivar pasture.

Methane concentration (mL/100 mL TGP) was higher for LS (17.3 and 16.4 mL/100 mL TGP for autumn and spring, respectively) than HS pasture (15.7 and 15.6 mL/100 mL TGP for autumn and spring, respectively), and in autumn than in spring (Table 4, Figure 2). Methane production (volume) did not vary with pasture type, nor season in any of the incubation times measured. There was a significant ‘pasture type’ × ‘season’ interaction for some methane production kinetics parameters, such as A, C, and n (Table 4). These interactions arose from greater A for LS pastures in autumn, whereas it was greater for HS pasture in spring, and the opposite trend was observed for C and n.

### 3.5. Microbial Nitrogen Yield and Nitrogen Utilisation

Ammonia N was 33.5% higher with LS pasture than with HS pasture, and 24.5% higher in autumn than in spring at both times of incubation (Table 5). NH_3_-N was 63.3% higher at 24 h of incubation than at 4 h. Nitrogen digestibility was similar between cultivars (*p* > 0.05), averaging 838 g/kg for HS and 789 g/kg for LS (Table 5). However, ND was 17.4% higher in autumn than in spring (*p* < 0.05). Microbial N was not affected by pasture type nor season. Estimated bacterial NUE was 18.5% higher with HS pasture than with LS and 14.1% higher in spring than in autumn (Table 5). The EMPS was not affected by pasture type and was 23.3% higher in spring than in autumn (Table 5).

## 4. Discussion

### 4.1. Chemical Composition and In Vitro Gas Production of Forage

The chemical composition of treatments is similar to values reported in autumn and spring for grass pastures in humid temperate regions [13,40]. However, the concentrations of WSC observed in this study for both cultivars, especially in the high sugar treatment (322 and 343 g/kg for autumn and spring, respectively) were greater than what is commonly reported for PRG pastures (74 to 243 g/kg) [41]. However, it must be noted that the current LS treatment was not ‘low sugar’ and, in comparison with other studies investigating high sugar grasses, was higher than most high sugar treatments [42]. The fact that, after harvesting, the samples were immediately frozen in the field and further freeze-dried could have reduced WSC losses due to respiration [43,44]. In the case of the LS cultivar (Extreme), the high WSC in spring may be due to its phenological stage, as it begins flowering earlier than the HS cultivar (Expo), and therefore increases its WSC [14]. As intended, the combination of cultivar selection and agronomic management (i.e., reduction in N fertilisation with a longer regrowth period) successfully modified chemical composition of forages, increasing the WSC and WSC:CP ratio and decreasing the CP and aNDF concentrations. Changes in chemical composition, such as the increase in WSC, the decrease in CP and NDF concentration with larger regrowth periods (from two to three leaves in terms of leaf appearance after defoliation) has been previously reported by Bryant et al. [45] and Loaiza et al. [14]. Similar effects have been observed when comparing low versus high N fertilisation levels [14,46]. As with other studies, cultivars selected for a high sugar concentration have lower CP and NDF to counter the elevated WSC concentration [45,47]. Additionally, in agreement with the literature, it was observed in our study that grass pastures typically have less WSC, more CP and NDF concentrations, but similar OM and NDF digestibility in autumn, compared with spring [40,48].

### 4.2. In Vitro Fermentation and Methane Output

The greater A and TGP observed for HS than LS was also reported by Lee et al. [47], which they related to the greater WSC and lower aNDF concentrations in the HS pasture improving total microbial fermentation. Although a higher WSC concentration is expected to result in a faster fermentation, this was not observed in this study as parameters of gas production rate were not affected. This is in accordance with Lovett et al. [25] and Keim et al. [49], who found no differences in the gas production rate among grass pastures in a vegetative stage of growth with a similar regrowth period, but found differences in CP and WSC. In the current study, LS in autumn had significantly higher hemicellulose, which may have accounted for a similar fermentation rate as the higher WSC of HS pasture. Whereas the difference in hemicellulose in spring was lost, the differential between WSC of the pastures was also smaller, which may have resulted in the comparable rates of fermentation. The difference in TGP may reflect the total carbohydrate content of the two grasses (WSC + aNDF) with comparable OMD and NDFD, which was higher for HS in both spring and autumn.

A similar pattern to TGP was observed for the concentration of tVFA, where HS tended to be greater than LS, as well as spring pastures compared with those harvested in autumn. Gas production represents fermentability of feedstuffs, and it is expected that an increase in TGP (and therefore fermentability) results in greater VFA production in the rumen [50]. A greater gas and VFA production has been previously reported for ryegrass varieties with greater WSC concentrations [24,47]. Further, it has been previously reported, as also observed here, that increasing the availability of WSC and decreasing the NDF concentration in grasses not only influences total VFA production, but also the molar proportions, with greater propionate and lower acetate, and therefore a lower acetate:proportionate ratio [8,47,51]. Such shifts reflect a more amylolytic microbial population, as observed with the supplementation of sucrose or inulin [8,52]. However, in the current study, the effect on propionate was not consistent, and depended on the incubation time and season of the year. The early fermentation (evaluated at four hours of incubation) resulted in greater relative proportion of tVFA for HS pastures in both autumn and spring, whereas, at 24 h of incubation, it was greater for HS in autumn without differences in spring. Niderkorn et al. [53] also observed that the acetate:propionate ratio is lower at earlier (3.5 h) than at later (24 h) sampling times. This may be related to differences in chemical composition of pastures across seasons. The large differences in WSC and aNDF in autumn (HS presented 98 g/kg more WSC and 77 g/kg less aNDF compared with LS) may have resulted in a consistent difference in propionate relative proportion of tVFA and acetate:propionate ratio at early (4 h) and late (24 h) fermentation. Whereas, in spring, the differential in WSC for HS was smaller (+50 g/kg WSC) with no significant differences in aNDF. Purcell et al. [24] reported similar results that when these differences in chemical composition (WSC and NDF) are small, it usually results in lack or only small effects on the extent of rumen in vitro fermentation and a lack of an effect on the relative proportions of the major fermentation VFAs produced. Differences in BCVFA among pasture types and season may be related to the greater CP concentration of pastures and lower total carbohydrate (reported above) harvested in autumn and LS compared to HS, as there is a strong positive correlation between CP level and isovalerate and valerate production [50]. Branched-chain VFAs are produced in the rumen from the deamination and decarboxylation processes from feed or microbial amino acids, especially with limited available energy [54]. Thus, the differences in BCVFA concentrations probably reflect the differences in one or both of these components [55].

The production of acetate and butyrate liberates hydrogen, whereas propionate serves as a net hydrogen sink. Consequently, diets that increase propionate and decrease acetate in the rumen are often associated with a reduction in ruminal CH_4_ production [5]. Stoichiometrically, methanogensis would be reduced when the ratio of acetate:propionate was reduced, as observed here [56]. Thus, it is expected that forages with higher WSC and a lower proportions of plant cell walls (lower OMD), would promote the production of propionate and reduce acetate in the rumen, thereby decreasing the amount of CH_4_ [57]. However, results in the literature are variable, for example Purcell et al. [24,58] and Keim et al. [49] observed no differences on in vitro CH_4_ from grasses with different concentrations of WSC and NDF. Whereas, Lovett et al. [25] and Purcell et al. [23] observed that increasing WSC in ryegrass reduced CH_4_ output, which is in agreement with the current study, for CH_4_ as a proportion of TGP. In autumn, the CH_4_ proportion of TGP was significantly greater for LS than for HS for only the first two hours of fermentation, but numerically higher throughout the whole incubation (Figure 2). Whereas, for spring the differences were less evident, as difference were smaller as already described above. This is in accordance with the interaction between time, pasture type and season for propionate proportion of tVFA, as already discussed. Hence, for reducing CH_4_ output from pastures, it is necessary to increase WSC considerably, and this must be accompanied by a reduction in ADF/NDF concentration, such as that which occurred in autumn. As in our study, the difference across the seasons of the year and regrowth periods are more consistent [24,25,58], and are the result of advancing maturity, with concomitant reductions in WSC concentration and lignification of plant cell walls. This promotes the production of acetate in the rumen from fibrolytic methanogenic microbial communities, and subsequently, increases the amount of CH_4_ produced per unit of forage digested [5].

### 4.3. Microbial Nitrogen Yield and Nitrogen Utilisation

Keim and Anrique [41] suggested that an effective approach for improving NUE in grazing systems was through a combination of different agronomic management, such as reducing N fertilisation, increasing grazing intervals, using selected cultivars with high sugar content, or adjusting the access to the pasture, according to the daily patterns of WSC and CP concentrations. In the current study the differences between HS and LS treatments were achieved by modifying N fertilisation, grazing interval, and cultivar selection. This resulted in differences in chemical composition that were mentioned above, and in terms of in vitro N metabolism, reduced NH_3_-N at both 4 h and 24 h of incubation and improved NUE. Coincidentally, cows grazing the same HS pastures that were sampled in autumn for this study presented a lower milk urea concentration, compared with the LS pasture, although the difference did not reach statistical significance [59]. However, there was no effect on microbial N yield, N digestibility, nor EMPS. As there was no increase in the microbial N yield or EMPS, it is expected that the reduction in NH_3_-N was more related to the lower CP concentration of HS pastures in both seasons, rather than due to a better balance between energy and N supply in the rumen increasing EMPS as observed by Lee et al. [8]. The current findings are in agreement with Chaves et al. [60] and Keim et al. [49], who found that the in vitro NH_3_-N production appears to be related to forage crude and soluble protein concentrations. It may, in the current study, mean that the CP concentration of HS and the subsequent in vitro rumen NH_3_-N released were limiting a response to a greater energy supply from the WSC concentration, and therefore no enhanced microbial N yield was observed. The EMPS values were on the low side of the previously reported values from whole forage diets [54,61], which may suggest a limited substrate supply or retardation of microbial protein synthesis through lack of pH regulation or intermediate build-up [8]. However, Satter and Slyter [62] reported that rumen NH_3_-N concentrations below 3.5 mmol/L retard microbial protein synthesis in vivo, which was below the values found in the current study. As reviewed by Keim and Anrique [41], NUE increased with lower CP contents, due to greater efficiency of the capture of rumen NH_3_ by the microbial population and/or the recycling of N through saliva into the rumen [54]. The NUE reported in this study were similar to those of Merry et al. [61] for a grass-red clover (*Trifolum pratense* L.) silage diet (65%), and Bach et al. [35] for a 50:50 orchard grass (*Dactylis glomerate* L.):legume pasture diet (64%), and higher than the values reported by Berthiaume et al. [63] for alfalfa (*Medicago sativa* L.: 41%), where the CP concentration was greater. Differences among seasons (autumn and spring) in terms of NH_3_-N and NUE are also explained by the differences in chemical composition (WSC and CP concentrations), as mentioned above.

As opposed to pasture type, significant effects of season were observed for ND and the EMPS. The lower ND observed in spring might be related to the phenological stage and the distribution of N associated with lignified tissue. Loaiza et al. [14] observed greater buffer-soluble proteins (A + B1) and lower buffer-insoluble, neutral detergent-soluble true protein (B2) crude protein fractions as a proportion of total CP in autumn compared with spring. The greater EMPS in spring was explained, as the microbial N yield was similar for both pastures with lower ND for spring pastures.

### 4.4. Implications

In the current study, strategies that can be routinely applied at a commercial farm level, combining genetics and management to elevate WSC and reduce CP, were evaluated using in vitro rumen fermentation techniques to determine the effect on NUE and CH_4_ production. As expected, pasture type differed in WSC and CP concentrations with HS averaging 332 g/kg DM across seasons, whilst LS averaged 259 g/kg; whereas LS had 30.4% more CP than HS. These changes in chemical composition modified in vitro rumen fermentation, tended to increase tVFA production, reducing the acetate:propionate ratio and CH_4_ production relative to TGP and improved NUE. Such responses could suggest a methodology for more sustainable grazing livestock systems by combining genetics and relatively simple management strategies. However, extrapolations of in vitro results to in vivo situations must be done carefully. For example, in the rumen itself, feed and microbial biomass are subject to passage, and VFA is subject to passage and absorption, thus, the mechanisms governing microbial efficiency and VFA molar proportions in vitro are not necessarily valid in vivo [64]. Moreover, both NUE and CH_4_ are affected by dry matter intake and ingestive behaviour [57,65]. In vivo studies have shown that both NUE [66,67] and CH_4_ production [68] can be improved by strategic pasture management. Therefore, in vivo studies with cattle are required to confirm the potential of these measures to increase the sustainability and to reduce the environmental impact of grazing livestock production systems. Additionally, a holistic approach must be considered, because an improvement in nutritional value must not be at the expense of pasture production, and therefore the ruminant grazing systems’ productivity; for example, Rivero et al. [69] found that high sugar grasses had lower annual dry matter productivity, and no preference was shown by grazing cattle.

## 5. Conclusions

The combination of ryegrass cultivar selection, delaying defoliation frequency to a three leaves per tiller stage of growth and reducing N fertilisation rates from 250 to 83.3 kg/ha N per year increases the average WSC by 28% and reduces CP by 30.4%. These changes in chemical composition modify in vitro rumen fermentation, tending to increase tVFA production, reduce the acetate:propionate ratio and CH_4_ production relative to TGP, and improve NUE through lower rumen NH_3_-N. In vivo studies with cattle are required to confirm the true potential of these measures to increase sustainability and to reduce the environmental impact of grazing livestock production systems.

## Figures and Tables

**Table 1:** Chemical composition and in vitro digestibility of pastures across regrowth periods.

	Autumn		Spring			*p*-Value		
Variable	HS	LS	HS	LS	SEM	P	S	P × S
WSC	322a	224c	343a	293b	10.3	***	**	n.s.
CP	154b	202a	120c	156b	4.58	***	***	n.s.
WSC to CP ratio	2.10b	1.11c	2.86a	1.88b	0.117	***	***	n.s.
aNDF	379b	456a	385b	391b	6.1	***	**	***
ADF	206b	227a	230a	238a	4.9	*	*	n.s.
Hem	173b	230a	156c	153c	4.2	***	***	***
Ash	84.8a	67.1b	71.6b	80.3a	3.34	*	**	n.s.
OMD	608	546	606	631	27.9	n.s.	n.s.	n.s.
tOMD	942	932	929	923	6.5	n.s.	n.s.	n.s.
NDFD	789	784	776	760	11.8	n.s.	n.s.	n.s.

HS: high sugar pasture; LS: standard cultivar pasture; SEM: standard error of the mean; P: pasture type; S: season; P × S: interaction between pasture type and season; WSC: water soluble carbohydrates (g/kg DM), CP: crude protein (g/kg DM); aNDF: neutral detergent fibre (g/kg DM); ADF: acid detergent fibre (g/kg DM); Hem; hemicellulose (aNDF–ADF; g/kg DM); OMD: apparent OM digestibility (g/kg); tOMD: true OM digestibility (g/kg); ND: nitrogen digestibility (g/kg); NDFD: neutral detergent fibre digestibility (g/kg); *p* *: *p* ≤ 0.05; **: *p* ≤ 0.01; ***: *p* ≤ 0.001; n.s.: not significant; Values on the same row followed by different letters present significant differences.

**Table 2:** Effect of pasture type and season on in vitro gas production kinetics.

	Pasture Type		Season			*p*-Value		
Variable	HS	LS	Autumn	Spring	SEM	P	S	P × S
TGP24	218	204	204	218	3.2	*	*	n.s.
PF	4.30	4.58	4.62	4.27	0.089	n.s.	*	n.s.
Predicted Michaelis–Menten parameters								
A	249	232	234	247	4.0	*	n.s.	n.s.
C	0.12	0.11	0.11	0.12	0.003	n.s.	n.s.	n.s.
MDR	0.12	0.12	0.12	0.13	0.003	n.s.	n.s.	n.s.
K	6.48	6.52	6.61	6.39	0.147	n.s.	n.s.	n.s.
n	149	148	148	149	0.02	n.s.	n.s.	n.s.
ta	3.11	3.1	3.15	3.06	0.079	n.s.	n.s.	n.s.
tb	13.6	13.7	13.9	13.4	0.35	n.s.	n.s.	n.s.
tc	28.4	28.9	29.3	28.0	0.93	n.s.	n.s.	n.s.

HS: high sugar pasture; LS: standard cultivar pasture; SEM: standard error of the mean; P: pasture type; S: season; P × S: interaction between pasture type and season; TGP: 24-h gas production (mL/g OM); PF: partitioning factor (digested OM per TGP); A: asymptotic gas production (mL/g OM); C: degradation rate at half-life (%/h); MDR: maximal degradation rate (%/h); n: shape of the curve; K; ta; tb; tc: time to produce 50, 25, 75 and 90% of gas production, respectively; *p* *: *p* ≤ 0.05; n.s.: not significant.

**Table 3:** Effect of pasture type, season, and time on in vitro volatile fatty acids concentration.

	4 h				24 h											
	Autumn		Spring		Autumn		Spring			*p*-Value						
Variable	LS	HS	LS	HS	LS	HS	LS	HS	SEM	P	S	P × S	T	T × P	T × S	T × P × S
VFA concentration (mM)																
Total VFA	30.7	32.9	37.1	38.3	58.0	62.7	72.3	74.2	1.95	†	***	n.s.	***	n.s.	*	n.s.
Acetate	21.0	22.1	26.2	24.8	39.1	40.6	47.1	47.7	1.60	n.s.	***	n.s.	***	n.s.	n.s.	n.s.
Propionate	7.06	8.05	8.61	10.8	12.7	15.5	17.7	19.3	0.514	***	***	n.s.	***	n.s.	**	n.s.
Butyrate	1.54	1.60	1.41	1.87	4.21	4.85	5.07	5.88	0.185	**	**	n.s.	***	n.s.	**	n.s.
BCVFA	1.08	1.18	0.91	0.86	1.93	1.65	1.73	1.41	0.090	**	***	n.s.	***	n.s.	n.s.	n.s.
Acetate	68.4	67.0	70.2	64.7	67.5	64.8	65.2	64.3	1.07	***	n.s.	n.s.	†	n.s.	n.s.	n.s.
Propionate	23.0	24.5	23.4	28.1	21.9	24.8	24.4	25.9	0.58	***	***	n.s.	n.s.	n.s.	n.s.	**
Butyrate	5.02	4.81	3.90	4.91	7.26	7.76	7.00	7.93	0.432	n.s.	n.s.	n.s.	***	n.s.	n.s.	n.s.
BCVFA	3.52	3.68	2.48	2.30	3.33	2.65	2.40	1.90	0.319	†	***	n.s.	n.s.	n.s.	n.s.	n.s.
A:P	3.00	2.74	3.05	2.30	3.08	2.61	2.67	2.48	0.121	**	*	n.s.	n.s.	n.s.	n.s.	†
(A + B):P	3.19	2.93	3.21	2.48	3.41	2.92	2.96	2.78	0.112	***	*	n.s.	n.s.	n.s.	n.s.	*

HS: high sugar pasture; LS: standard cultivar pasture; SEM: standard error of the mean; P: pasture type; S: season; P × S: interaction between pasture type and season; T: time of incubation; T × P: interaction between time and pasture type; T × S: interaction between time and season; T × P × S: interaction between time, pasture type and season; VFA: volatile fatty acids; BCVFA: isobutyrate + isovalerate + valerate; A:P: acetate: propionate ratio; †: *p* ≤ 0.1; *p* *: *p* ≤ 0.05; **: *p* ≤ 0.01; ***: *p* ≤ 0.001; n.s.: not significant.

**Table 4:** Effect of pasture type and season on in vitro methane (CH_4_) output.

	Autumn		Spring			*p*-Value		
Variable	HS	LS	HS	LS	SEM	P	S	P × S
CH_4_ (mL/100 mL TGP)	15.7c	17.3a	15.6c	16.4b	0.13	***	*	n.s.
CH_4_ production at 6 h (mL)	16.4	16.2	17.0	17.1	1.13	n.s.	n.s.	n.s.
CH_4_ production at 12 h (mL)	25.3	24.5	25.6	25.8	1.70	n.s.	n.s.	n.s.
CH_4_ production at 24 h (mL)	33.2	33.4	34.9	34.1	0.65	n.s.	n.s.	n.s.
Predicted Michaelis–Menten parameters for CH_4_ production								
A	43.8b	59.5ab	61.8a	51.0ab	3.37	n.s.	n.s.	*
C	0.069a	0.038b	0.037b	0.057ab	0.0046	n.s.	n.s.	**
K	9.40	20.6	18.5	13.0	2.75	n.s.	n.s.	n.s.
n	1.22a	0.98b	0.99b	1.11ab	0.044	n.s.	n.s.	*
ta	3.81	6.20	5.72	4.46	0.622	n.s.	n.s.	n.s.
tc	23.0	69.5	60.4	38.4	11.57	n.s.	n.s.	n.s.

HS: high sugar pasture; LS: standard cultivar pasture; SEM: standard error of the mean; P: pasture type; S: season; P × S: interaction between pasture type and season; TGP: total gas production; A: asymptotic methane production (mL/g OM); C: degradation rate at half-life (h^−1^); n: shape of the curve; K; ta; tc: time to produce 50, 25 and 75% of methane production, respectively; *p* *: *p* ≤ 0.05; **: *p* ≤ 0.01; ***: *p* ≤ 0.001; n.s.: not significant; Values on the same row followed by different letters present significant differences.

**Table 5:** Effect of pasture type and season on in vitro nitrogen metabolism and efficiency.

	Pasture Type		Season			*p*-Value		
Variable	HS	LS	Autumn	Spring	SEM	P	S	P × S
NH_3_-N4h (mmol/L)	6.11	8.16	7.92	6.36	0.410	**	**	n.s.
NH_3_-N24h (mmol/L)	10.4	12.9	13.2	10.1	0.410	**	**	n.s.
ND (g/kg N)	780	838	877	750	30.4	n.s.	*	n.s.
MN (mg/g OM)	14.6	15.3	15.2	14.7	0.53	n.s.	n.s.	n.s.
NUE (%)	64.1	54.1	55.2	63.0	2.21	*	*	n.s.
EMPS (g MN/kg of truly digested OM)	22.1	20.9	19.3	23.8	1.30	n.s.	*	n.s.

HS: high sugar pasture; LS: standard cultivar pasture; SEM: standard error of the mean; P: pasture type; S: season; P × S: interaction between pasture type and season; NH_3_-N: ammonia nitrogen (mM); ND: nitrogen digestibility (g/kg); MN: microbial nitrogen (mg/g OM); NUE: nitrogen use efficiency (%); EMPS: efficiency of microbial protein synthesis (g MN/kg of truly digested OM); *p* *: *p* ≤ 0.05; **: *p* ≤ 0.01; n.s.: not significant; Values on the same row followed by different letters present significant differences.
